# Exploring the impact of ‘hostile environment’ policies on psychological distress of ethnic groups in the UK: a differences-in-differences analysis

**DOI:** 10.1007/s00127-024-02705-2

**Published:** 2024-07-08

**Authors:** K. Dotsikas, M. McGrath, D. P. J. Osborn, K. Walters, J. Dykxhoorn

**Affiliations:** 1 Division of Psychiatry, UCL, Maple House, 149 Tottenham Court Road, London, W1T 7NF UK; 2 Equipe de Recherche en Epidémiologie Sociale, Sorbonne Université, INSERM, Institut Pierre Louis d’Epidémiologie Et de Santé Publique, 75012 Paris, France; 3 Discipline of Psychiatry and Mental Health, University of New South Wales, Sydney, 2052 Australia; 4 Camden and Islington NHS Foundation Trust, London, NW1 OPE UK; 5 Department of Primary Care and Population Health, UCL, Royal Free Campus, London, NW3 2PF UK

**Keywords:** Common mental disorders, Depression, Anxiety, Psychiatric epidemiology, Temporal trends, Difference-in-differences, Ethnic minoritised groups

## Abstract

**Purpose:**

In 2012, the UK government announced legislation changes and heightened immigration controls designed to create a ‘hostile environment for illegal migration.’ We measured changes in psychological distress among people from minoritised ethnic groups compared to White British controls before and throughout the implementation of these policies.

**Methods:**

We used the UK Household Longitudinal Survey to estimate difference-in-difference models for six ethnic groups (Bangladeshi, African, Caribbean, Indian, Pakistani, and White British) in three eras: pre-policy (2009–2012); (2) transition (2012–2016); and (3) ongoing policy (2016–2020). We calculated the adjusted marginal mean psychological distress score at each era using the 12-item General Health Questionnaire (GHQ).

**Results:**

In the pre-policy era, we found higher psychological distress for the Pakistani, Bangladeshi, and Caribbean groups compared to the White British group. We observed patterns consistent with increasing psychological distress during the transition era for the Pakistani and Bangladeshi groups, with further increases in the ongoing era for the Bangladeshi group. Levels of psychological distress the Indian and African groups were similar to the White British group in the pre-policy era and decreased over successive eras. A small decrease was observed in the Caribbean group across policy eras, while levels remained stable in the White British group.

**Conclusion:**

We found evidence that psychological distress increased among Pakistani and Bangladeshi individuals following the introduction of hostile environment policies but did not detect increased distress in other ethnic groups. This finding underscores the importance of disaggregating analyses by ethnic group to capture the distinct experiences.

**Supplementary Information:**

The online version contains supplementary material available at 10.1007/s00127-024-02705-2.

## Introduction

In 2012, then Home Secretary Theresa May announced a desire ‘to create here in Britain a really hostile environment for illegal migration’, which has later been referred to by the Government as the ‘compliant environment policy’. The passage of the Immigration Acts of 2014 and 2016 enacted this ‘hostile environment’ by implementing several policy changes that aimed to increase social exclusion for undocumented migrants. Alongside these policy changes, other initiatives began such as the ‘Go Home vans’, a 2013 government campaign in which vans with advertising slogans reading ‘In the UK illegally? Go home or face arrest’ drove around six London boroughs with high concentrations of immigrants and minoritised ethnic individuals. Further, anti-migrant rhetoric increased in the lead up to the 2016 Brexit referendum, and a spike in racist or religious abuse hate crimes reported to police was observed in the period directly following the referendum, with evidence supporting a causal link with the referendum [1].

The enforcement of national borders was extended beyond traditional means to dissuade illegal residence in the UK and prevent migrants from accessing basic services, employment opportunities, or housing. Griffiths and Yeo describe this process as the ‘deputisation’ of immigration control, wherein the responsibility of enacting immigration policy is diffused to a range of actors, including public servants, medical practitioners, private companies, and ‘ordinary people’ [2]. Within the health system, identification checks were introduced for patients accessing non-emergency hospital care, and a memorandum of understanding between the Department of Health, National Health Service, and the Home Office allowed for the sharing of non-clinical data ‘to support effective immigration enforcement’ [3, 4]. Further, the Immigration Act 2016 placed immigration control in the hands of employers and landlords, making them criminally responsible for employing or renting to someone that they have reasonable cause to believe is not permitted to work or reside in the UK [2]. Such deputisation leads to third parties conducting discriminatory checks and employing restrictions based on ethnicity as they seek to avoid sanctions, negatively impacting both migrants and lawful UK residents from minority ethnic backgrounds [5]. In this way, the hostile environment enforces structural racism by encouraging racial profiling through discriminatory checks against anyone ‘foreign-looking’ [2]. Indeed, ethnic minoritised groups have been disproportionately subject to immigration checks and denied access to basic services; for example, evidence indicates that since the advent of the hostile environment policies, landlords are less likely to rent to people who are foreign nationals or ethnic minoritised individuals without British passports [2, 5–7].

By 2018, the Government acknowledged the failings of the hostile environment policies. While these policies initially targeted undocumented and irregular migration, they impacted members of ethnic minoritised groups, including those with the legal right to reside in the UK.

This widespread structural discrimination faced by UK residents belonging to ethnic minoritised groups in the last ten years poses a risk to their mental health (Qureshi et al. 2020); people belonging to ethnic minority groups in the UK who have reported experiences of perceived racial discrimination are more likely to have greater psychological distress [8–10], with evidence of a cumulative effect [11]. Exposure to individual and structural discrimination directly impacts mental health by acting as a chronic psychosocial stressor, activating the biological stress response [12]. Structural racism additionally indirectly impacts mental health by unjustly allocating societal resources on the basis of ethnicity, leading to material insecurity and further stress [13].

Ethnic groups are not homogenous, and those belonging to ethnic minoritised groups represent a diverse population with varied experiences of interpersonal, structural, and institutional racism. These differing experiences of racism are likely to result in a unique relationship with mental health problems between ethnic groups. Nevertheless, there is evidence that the mental health of certain ethnic groups in the UK is already worse than White British people [14]; for example, common mental disorders are more prevalent among Black women [15]. In light of the hostile environment and increases in racial discrimination, these mental health disparities may widen. However, to date, most research on racism and mental health has taken place in the US, and no study has examined the population impact of the hostile environment on the mental health of ethnic minoritised groups in the UK.

*Aims:* In this study, we aimed to measure changes in psychological distress among people from ethnic minoritised groups compared to White British controls following the introduction of hostile environment policies. We hypothesised that we would see an increase in psychological distress over the study period, particularly in minoritised ethnic groups.

## Methods

### Data source

We used data from Understanding Society, the UK Household Longitudinal Survey (UKHLS), which collects information annually. Approximately 40,000 households were sampled at Wave 1 (2009–2010) through a stratified, clustered equal probability sampling process. Postcode sectors were randomly selected from geographical strata as the primary sampling unit (PSU), and households were randomly selected from the PSUs [16]. UKHLS incorporated an additional Ethnic Minority Boost (EMB) sample at Wave 1 by sampling addresses in areas with a high concentration of important ethnic minoritised groups in order to provide at least 1000 adult interviews from these groups. We used data collected from adults (ages 16 or older) in the main and EMB samples who identify as belonging to one of the following six ethnic groups: Bangladeshi, Black African, Black Caribbean, Indian, Pakistani, and White British [16]. Participants were followed over 10 successive data collection waves of UKHLS covering the years 2009–2020 (Wave 1 through Wave 10). We excluded participants from Northern Ireland, as they did not contribute to the EMB. We excluded participants whose interviews were completed by a proxy respondent in Wave 1. When a participant was not able to complete the interview, another member of their household (e.g. partner or adult children) may complete the interview on their behalf as a proxy respondent. The information provided by proxy respondents was focused on reporting factual information but does not include questions about the mental health of the respondent.

### Exposure

The primary exposure in this study was exposure to hostile environment policies, which were introduced in the UK between 2012 and 2016. We divided the follow-up time into three eras: (1) pre-hostile environment (2009–2012, including Waves 1–3); (2) transition era (2012–2016, including Waves 4–6); and (3) ongoing hostile environment policies (2016–2020, including Waves 7–9).

### Outcome

Self-reported symptoms of psychological distress were measured using the General Health Questionnaire (GHQ-12). This 12-item questionnaire asks about happiness, symptoms of depression and anxiety, and sleep disturbance and is a well-validated tool for estimating psychological distress [17]. GHQ-12 scores were calculated at each wave of UKHLS using the standard GHQ-12 scoring method, where the four response options for each item were dichotomised and summed across all items to create a continuous score, where a higher score indicated more psychological distress [18].

### Covariates

We considered several covariates in our analysis, including age (continuous), sex (female; male), marital status (partnered; separated/divorced/widowed; single), educational qualifications (A-levels or other post-secondary qualifications; GCSC or high school diploma or lower), citizenship status (British citizen; not British citizen), and urban or rural residence. We took the measures of these covariates from each wave of UKHLS to take into account their time-varying nature, with the exception of sex, which we considered to be time-stable.

### Analysis

We described the sample characteristics at baseline (Wave 1) by ethnic group. We used difference-in-difference (DiD) models to estimate the effect of the hostile environment on mental health by comparing the change in mental health before and after the introduction of these policies in ethnic minoritised groups (treated group) compared to the White British group (control). DiD models are widely used to estimate the causal effects of an intervention in longitudinal observational data. This DiD model allowed us to estimate the potential treatment effect (hostile environment policies) on the outcome (mental health) by comparing change before, during, and after the introduction of these policies on the treated group (minoritised ethnic groups) compared to the control group (White British group).

We estimated used a linear mixed effects model for a continuous response. We used a likelihood ratio test to compare the model fit of a model with and without the interaction term between policy era and ethnic group, including the interaction term if there was evidence for improved model fit with the inclusion of an interaction term between era and ethnicity to estimate the relationship between ethnic group and mental health during the three eras of policy implementation. The interaction term tested the effect of being a member of a particular ethnic minoritised group in one of the two intervention policy periods, compared to being White British in the pre-policy period. Mixed models allowed us to account for our longitudinal data, with the waves of UKHLS clustered within each individual over time; we included a random intercept for each individual. We estimated the random effect at the individual level allowed us to account for the within-individual correlation of GHQ scores over the study period. The mixed effects model estimated fixed effects coefficients for the effect of era, ethnicity, and the interaction between era and ethnicity. The model also generated two random-effects parameters related to the clustering at the individual level: one which estimated the variance of individual-level errors, and the second to estimate the variance of errors within individuals (the variance of the residuals). The analysis was weighted at the level of the individual to account for sampling probability, differential non-response, and sampling error.

We calculated the marginal mean GHQ-12 score by ethnic group for each of the three policy periods. The marginal mean of GHQ is the predicted mean based on the mixed model if all the observations were treated as if they were fixed at a particular level of ethnicity and policy period; for example, the marginal mean of GHQ for the Bangladeshi group in the pre-policy period is the predicted mean GHQ if all observations in the cohort were treated as if they were Bangladeshi and in the pre-policy period. The values of the covariates were left as observed for the calculation of marginal mean scores.

**Fig. 1:**
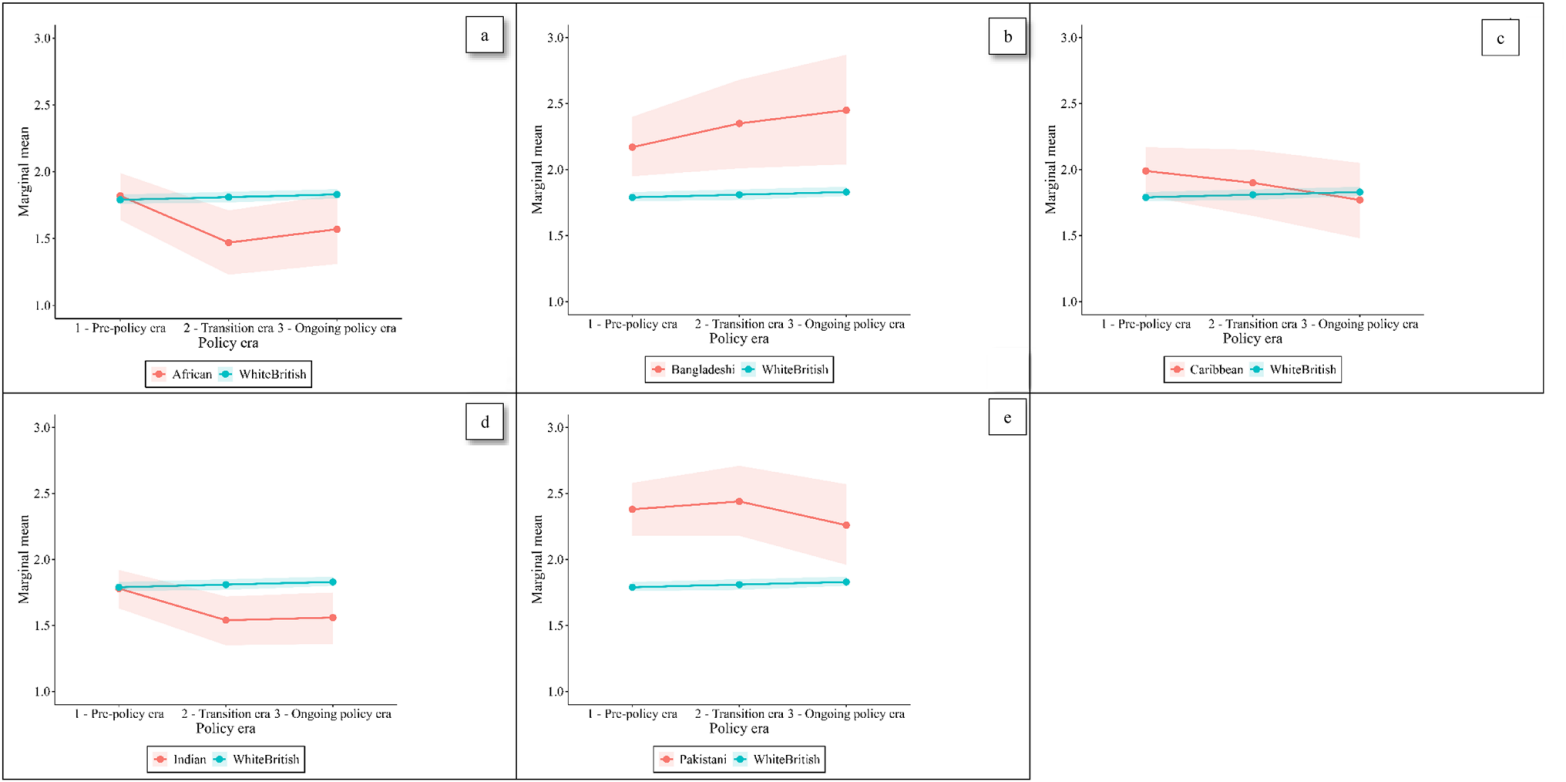
**a**–**e** Marginal mean psychological distress scores by ethnic group and policy era, imputed

We explored patterns of missingness in the data, including wave non-response patterns and item non-response for outcomes and covariates (Supplement A). We estimated inverse probability weights to account for differential non-response as part of our weighting strategy. We compared the sample characteristics of those who had complete data and those who had missing data on one or more variables. The data was assumed to be missing at random, and we used multiple imputation with chained equations to impute missing outcome and covariate data. Our primary outcomes were based on 100 imputed datasets. We conducted a sensitivity analysis on those with complete data (Supplement B). We used Stata 17.0 (MP-parallel edition) to conduct all analyses. We used R 4.3.2 to generate Fig. 1. Code for this analysis has been deposited on GitHub (https://github.com/lovelyoutliers/hostile_environment).

## Results

### Descriptive statistics

There were 42,968 participants included in our sample, most of whom were White British (n = 35,918; 83.6%). All other ethnic groups had between 1132 participants (Bangladeshi) and 1,905 participants (Indian). There were more females than males in our sample, which was most pronounced in among Caribbean (60.1% female), White British (56.4% female), and African (57.7% female) participants. In contrast, there was nearly equal proportions of females and males in the Bangladeshi (49.2% female) and Indian (48.7% female) participants (Table 1).

**Table 1:** Sample characteristics by ethnic group in Wave 1 (n = 42,968)

Sample characteristics	African		Bangladeshi		Caribbean		Indian		Pakistani		White British	
	n	%	n	%	n	%	n	%	n	%	n	%
Total												
n (% of total sample)	1438	3.3	1132	2.6	1137	2.6	1905	4.4	1438	3.3	35,918	83.6
Sex												
Female	830	57.7	557	49.2	683	60.1	927	48.7	759	52.8	20,253	56.4
Male	608	42.3	575	50.8	454	39.9	978	51.3	679	47.2	15,665	43.6
Age												
Mean (SD)	36.2	12.8	33.8	12.8	45.8	16.9	39.5	15.4	35.9	14.2	48.4	18.4
Education												
A-levels or higher	876	60.9	450	39.8	448	39.4	1,158	60.8	623	43.3	13,756	38.3
GCSE or lower	560	38.9	680	60.1	687	60.4	745	39.1	815	56.7	22,138	61.6
Missing	2	0.1	2	0.2	2	0.2	2	0.1	0	0.0	24	0.1
British citizen												
Yes	858	59.7	909	80.3	1036	91.1	1,384	72.6	1171	81.4	35,852	99.8
No	579	40.2	222	19.6	101	8.9	520	27.3	267	18.6	61	0.2
Missing	2	0.1	1	0.1	0	0.0	1	0.0	0	0.0	5	0.0
Urban/rural												
Urban	1430	99.4	1126	99.5	1131	99.5	1876	98.5	1433	99.6	26,648	74.2
Rural	8	0.6	6	0.5	6	0.5	29	1.5	5	0.4	9,270	25.8
Marital status												
Partnered/married/cohabiting	664	46.2	716	63.2	420	36.9	1,265	66.4	946	65.8	22,787	63.4
Separated/divorced/widowed	199	13.8	84	7.4	222	19.5	138	7.2	143	9.9	5,877	16.4
Single	575	40.0	331	29.2	493	43.4	502	26.4	348	24.2	7,244	20.2
Missing	0	0.0	1	0.1	2	0.2	0	0.0	1	0.1	10	0.0
Psychological distress (GHQ-12)												
Mean (SD)	1.86	2.81	2.24	2.94	2.01	2.95	1.78	2.85	2.22	3.19	1.73	2.87

*n* number, *SD* standard deviation, *GHQ* General Health Questionnaire

There were interesting patterns of education across the ethnic groups. In the African and Indian groups, over 60% had completed A levels or higher, with only 38.9% (African) and 39.1% (Indian) leaving school with GCSE qualifications or lower. These proportions were the inverse of what was seen in the White British, Bangladeshi, and Caribbean groups, where less than 40% achieved post-secondary qualifications (A levels or higher).

The proportions who had British citizenship also varied across ethnic groups. 99.8% of the White British group reported being a citizen of the UK. Several other ethnic groups had high proportions of citizens, including 91.1% of Caribbean participants, 81.4% of Pakistani participants, and 80.3% of Bangladeshi participants. The lowest proportion of citizenship was observed in African participants, with 59.7% reported having citizenship in the UK. Over 98% of participants from African, Bangladeshi, Caribbean, Indian, and Pakistani groups lived in urban areas. There was a smaller proportion of the White British living in urban areas (74.2%). Over 60% of participants in Bangladeshi, Indian, Pakistani, and White British groups were married or cohabitating. There were lower rates of marriage/cohabitation in the African (46.2%) and Caribbean (36.9%) groups.

At the start of the study, levels of psychological distress were highest in the Bangladeshi (mean GHQ: 2.24, SD: 2.94) and Pakistani (mean GHQ: 2.22, SD: 3.19) groups. The lowest levels of psychological distress were observed in the White British group (mean GHQ: 1.73, SD: 2.87) and Indian group (mean GHQ: 1.78, SD: 2.85). Mean GHQ scores changed within each ethnic group and by study wave (Supplement C).

### Missing data

There were two mechanisms of missing data in our study: wave non-response and item non-response. There were 42,968 participants in Wave 1, and each subsequent wave had reducing proportions of respondents. In the final wave of the study, only 38.3% of the original sample responded (Supplement A). Among respondents within each wave, there was missing data for confounders and outcomes (item non-response). There was less than 1% missing across all covariates in each wave, but there was a larger proportion of missing for psychological distress, ranging from 16.1% missing in Wave 1 to 4.0% in the later waves. We compared the characteristics of participants with complete data and participants with missing data. Participants were more likely to have missing data if they were from a minoritised ethnic group, had lower education, were not a British citizen, lived in an urban area, or were not in a marriage or cohabitation partnership (Supplement A).

### Difference-in-differences models

We used difference-in-difference (DiD) models to estimate the psychological distress and marginal mean marginal scores each ethnic group during the three policy eras: pre-policy era, transition era, and ongoing policy era.

### Linear mixed effects model

We found evidence of improved model fit when the interaction term was included (chi-squared value of 40.2 with 10 degrees of freedom). The adjusted linear mixed effects models found evidence for higher GHQ-12 scores in the pre-policy era among Pakistani (coef. 0.59, 95% CI 0.39–0.76), Bangladeshi (coef. 0.38, 95% CI 0.16–60), and Caribbean (coef. 0.19, 95% CI 0.00–0.38) groups compared to the White British group (Table 2).

**Table 2:** Unadjusted and adjusted linear mixed model, imputed^1^ (n = 42,968)

		Unadjusted				Adjusted^**1**^			
		Coeff	95% CI		p-value	Coeff	95% CI		p-value
Era									
Pre-policy era (ref.)									
Transition era		0.00	− 0.04	0.03	0.94	0.02	− 0.02	0.05	0.36
Ongoing policy era		0.02	− 0.02	0.06	0.28	0.04	0.00	0.08	0.04
Ethnicity									
African		0.28	0.09	0.46	< 0.01	0.02	− 0.15	0.20	0.79
Bangladeshi		0.51	0.26	0.76	< 0.001	0.38	0.16	0.60	0.00
Caribbean		0.36	0.17	0.56	< 0.001	0.19	0.00	0.38	0.05
Indian		0.12	−0.04	0.28	0.13	−0.02	− 0.17	0.13	0.81
Pakistani		0.79	0.58	1.00	< 0.001	0.59	0.39	0.79	0.00
White British (ref.)									

Interaction		Unadjusted				Adjusted^**2**^			
Era	Ethnicity	Coeff	95% CI		p-value	Coeff	95% CI		p-value
Transition	African	-0.34	-0.57	-0.11	< 0.01	-0.36	-0.59	-0.13	<0.01
	Bangladeshi	0.20	-0.17	0.57	0.30	0.16	-0.18	0.49	0.37
	Caribbean	-0.04	-0.29	0.21	0.75	-0.10	-0.35	0.14	0.41
	Indian	-0.18	-0.36	-0.01	0.04	-0.26	-0.44	-0.08	0.01
	Pakistani	0.08	-0.19	0.34	0.57	0.04	-0.23	0.32	0.75
Ongoing policy	African	-0.22	-0.51	0.07	0.13	-0.29	-0.55	-0.02	0.03
	Bangladeshi	0.30	-0.14	0.74	0.17	0.24	-0.23	0.70	0.31
	Caribbean	-0.15	-0.44	0.14	0.31	-0.26	-0.55	0.04	0.09
	Indian	-0.22	-0.43	0.00	0.05	-0.26	-0.46	-0.06	0.01
	Pakistani	-0.14	-0.45	0.17	0.38	-0.16	-0.48	0.16	0.33
Constant									
	Fixed effect	1.82	1.79	1.85	0.00	2.09	2.00	2.18	<0.001

			Unadjusted				Adjusted^**2**^		
Random-effects parameters^3^			Coeff	95% CI	RSE		Coeff	95% CI	RSE
Variance of individual-level errors		2.02	1.99	2.04	0.01	2.16	2.13	2.19	0.01
Variance of residuals (within-individual)		2.21	2.19	2.22	0.01	2.12	2.10	2.14	0.01

*Coef* coefficient, *CI* confidence interval, *Ref* reference category, *RSE* robust standard error

^1^100 imputed datasets

^2^Adjusted for sex, age, marital status, education, citizenship status, urban/rural status

^3^Mixed models with random effects to account for clustering within each individual over time

During the transition era, we observed decreased psychological distress scores in the African group (coef. − 0.36, 95% CI − 0.59 to − 0.13) and the Indian group (coef. − 0.26, 95% CI − 0.44 to − 0.08) compared to the pre-policy era. Lower levels of psychological distress were also seen in the ongoing policy era in the African and Indian groups, when compared to the pre-policy level. Similar patterns were observed in the complete case analysis (Supplement B).

### Marginal means

During the pre-policy era, the highest marginal mean level of psychological distress was found in the Pakistani group (adjusted coef. 2.38, 95% CI 2.18–2.58), which was higher than the level found in the White British group (coef. 1.79, 95% CI 1.76–1.83) (Table 3). Psychological distress was also higher in the Bangladeshi group (coef. 2.17, 95% CI 1.95–2.40) when compared to the White British group (Fig. 1). The Caribbean group had elevated psychological distress compared to the White British group in unadjusted models, but this was partially attenuated following adjustment (coef. 1.99, 95% CI 1.80–2.17).

**Table 3:** Marginal mean psychological distress scores by ethnic group and policy era, imputed^1^ (n = 42,968)

	Pre-policy era			Transition era			Ongoing policy era		
	Coeff	95%	CI	Coeff	95%	CI	Coeff	95%	CI
African									
Unadjusted	2.10	1.92	2.28	1.76	1.52	2.00	1.90	1.63	2.17
Adjusted^2^	1.82	1.64	1.99	1.47	1.23	1.71	1.57	1.31	1.83
Bangladeshi									
Unadjusted	2.33	2.08	2.58	2.53	2.16	2.89	2.66	2.27	3.05
Adjusted^2^	2.17	1.95	2.40	2.35	2.01	2.68	2.45	2.04	2.87
Caribbean									
Unadjusted	2.19	1.99	2.38	2.14	1.89	2.39	2.06	1.78	2.34
Adjusted^2^	1.99	1.80	2.17	1.90	1.65	2.15	1.77	1.48	2.05
Indian									
Unadjusted	1.94	1.79	2.10	1.76	1.57	1.94	1.75	1.55	1.96
Adjusted^2^	1.78	1.63	1.92	1.54	1.35	1.72	1.56	1.36	1.75
Pakistani									
Unadjusted	2.61	2.40	2.82	2.69	2.42	2.95	2.49	2.20	2.78
Adjusted^2^	2.38	2.18	2.58	2.44	2.18	2.71	2.26	1.96	2.57
White British									
Unadjusted	1.82	1.79	1.85	1.82	1.78	1.86	1.84	1.80	1.88
Adjusted^2^	1.79	1.76	1.83	1.81	1.77	1.85	1.83	1.80	1.87

*Coef* coefficient, *CI* confidence interval

^1^100 imputed datasets

^2^Adjusted for sex, age, marital status, education, citizenship status, urban/rural status

^3^Mixed models with random effects to account for clustering within each individual over time

During the transition era, the marginal mean psychological distress score increased for the Pakistani and Bangladeshi groups (coef. 2.44, 95% CI 2.18–2.71; coef. 2.35, 95% CI 2.01–2.69 respectively), remaining higher than the White British group (coef. 1.81, 95% CI 1.77-1.85). The remaining ethnic groups saw their psychological distress scores remain stable or decrease, with the greatest decrease seen in the African group (coef. 1.47, 95% CI 1.23–1.71) and Indian group (coef. 1.54, 95% CI 1.35–1.72), which were both lower than the level in the White British group. A reduction was also observed in the Caribbean group, from 1.99 (95% CI 1.80–2.71) to 1.90 (95% 1.65–2.51).

In the ongoing policy era, the marginal mean psychological distress score continued to rise for the Bangladeshi group, reaching 2.45 (95% CI 2.04–2.87). Psychological distress reached its lowest level in the Pakistani group during the ongoing policy period (coef. 2.26, 95% CI 1.96–2.57), but remained higher than the mean distress in the White British group (1.83, 95% CI 1.80–1.87). There was a slight increase in the marginal mean score in the African group (coef. 1.57, 95% CI 1.31–1.83), overlapping with the estimates for the White British group. In the Indian group, psychological distress level remained lower than the White British group in the ongoing policy era (coef. 1.56, 95% CI 1.36–1.75), and we observed a further reduction in the level of psychological distress in the Caribbean group (coef. 1.77, 95% CI 1.48–2.05).

## Discussion

In this first quantitative assessment of the potential impact of the hostile environment policies on the mental health of different ethnic groups in the UK; we found that the hostile environment was associated with negative impact on the mental health of Pakistani and Bangladeshi groups. These minoritised ethnic groups experienced greater psychological distress during and after the implementation of the hostile environment policies, compared to the pre-policy period and compared to the White British group, although the effect sizes were modest. We found no evidence of negative impact on mental health for the Indian, African, and Caribbean groups. There was no overall common effect of the hostile environment on psychological distress across the six ethnic groups included in our study. This finding underlines the importance of considering ethnic groups not as a monolith, but instead as groups with distinct identities and relationships with mental health.

The differences in the mental health impact of the hostile environment between the six ethnic groups may be partially explained by intersections of ethnicity with other socioeconomic factors. We found the highest rates of psychological distress among Pakistani and Bangladeshi people. Pakistani and Bangladeshi people had the lowest household incomes, employment rates, and median gross hourly pay among ethnic groups in the UK [19]; long-term trends show that this income inequality for Pakistani and Bangladeshi people has persisted over the last 15 years [20]. Reflecting our findings, Bamford et al. (2021) similarly found the highest odds of psychological distress among Pakistani people in a study on ethnicity and mental health in the UK during the hostile environment transition era [21]. They examined different risk factors and found that gender, economic insecurity, and the length of settlement in the UK may play a role in the mental health of different ethnic groups [21]. Similarly, Wallace et al. (2016) found that the poor mental health of Pakistani and Bangladeshi people in the UK during the same time period was partially explained by racial discrimination and socioeconomic disadvantage [11]. The pre-existing economic insecurity faced by Pakistani and Bangladeshi people in the UK may mean they have limited resources to weather potential increased insecurity due to hostile environment policies.

This analysis identified some potential differences in psychological distress by ethnic group following the introduction of new immigration policies in the UK. However, it should be noted that assessing the causal relationship between these policies and mental health would require detailed consideration of the measurement of the exposure over time. The hostile environment is in fact a collection of several different policies, which each might have different and perhaps opposing relationships on the different ethnic groups included in this study. For example, data from 2016 to 2018 shows that Indian households were the most likely to be owner occupiers out of all ethnic groups in England, suggesting that a relatively low proportion of Indian people have been subject to discrimination in the private rental sector [22]. Pakistani, Bangladeshi, Black African and Black Caribbean people are more likely to experience insecure work, indicating that they may face greater job insecurity in light of discriminatory employment restrictions [23]. We were surprised that we did not detect an increase in psychological distress among the Caribbean group in our sample, as they were subjected to targeted efforts by the Home Office to deport individuals without documentation which demonstrated their legal right to remain in the UK, later called the Windrush Scandal [26]. The high proportion of Caribbean participants in UKHLS who reported British citizenship could partially account for this, if many participants did not feel as though the immigration policy changes affected them due to their citizenship status. The unexpected findings in the Caribbean group further highlight the challenge of estimating causal effects of broad policy change on mental health of population subgroups, which may require novel methodological approaches to consistently disentangle the complete effects of policy change on mental health. The heterogeneity of hostile environment experiences underscores the importance of investigating minoritised ethnic groups separately.

The 12-item General Health Questionnaire (GHQ) is a multidimensional measure of mental health, which includes anxiety and depression, which are relevant to assessing the impact of hostile environment policies. However, there are some limitations with using this measure, particularly in sensitivity to measure change over time. In our study, the White British group showed no meaningful change in GHQ scores over the decade of follow-up, suggesting the measure may not be sensitive to change over extended time periods. However, self-reported measures of mental health, including the GHQ, do present advantages for measuring mental health over alternative methods, such as health services records, which can underestimate mental health problems. Some ethnic groups are less likely to access mental health services due to barriers to accessing mental health services including stigma around mental health, language barriers, and experiences of discrimination in the health care system [24], reducing their rate of diagnosis. Nevertheless, future research may consider alternative outcome measures.

Effect sizes for GHQ also appear to be relatively small. However, a score of 2/3 has been found to be the most appropriate cut-off and is consistent with the diagnosis of a common mental disorder [17]; almost all of the ethnic groups in our study had a mean GHQ score over two across the three eras, and the Pakistani and Bangladeshi groups had GHQ scores close to three during the transition and ongoing policy eras. Furthermore, while an increase of a few decimal points in a GHQ score may be inconsequential at the individual level, an increase of 0.33 points in the marginal mean GHQ (as seen in the Bangladeshi group) is equivalent to shifting the entire population of an ethnic group to a higher level of psychological distress, as described by Geoffrey Rose [25]. Even small changes in GHQ at the population level could indicate a meaningful increase in mental health problems experienced in that population group.

Our study was limited by relatively small sample sizes for the minoritised ethnic groups, reflected in the wide confidence intervals for our estimates. To take into account further heterogeneity within ethnic groups, such as stratification by gender, our sample size would be reduced, further limiting our ability to detect an effect. Future research needs to be conducted using surveys that robustly sample ethnic groups, as there are clear limitations to a sub-group analysis using a general population survey. The small sample sizes may explain why no meaningful effect of the hostile environment was detected for the Black Caribbean group. Considering the documented discrimination and deportation risk faced by this ethnic group linked to immigration policy change [26], this finding was surprising, and requires further investigation. We were also not able to disaggregate findings by migrant status as data on time since immigration was not available, and it is plausible that there may be distinct patterns of psychological distress based on migrant generation (e.g. immigrants and second-generation migrants).

Finally, we conducted a mixed effects model to account for the individual-level clustering across study waves. While we adjusted our DiD model using several time-varying covariates, our estimates are not necessarily reliable causal estimates for the effect of the hostile environment on ethnic groups if our assumption of no unmeasured confounding was violated, although the potential bias is reduced in the presence of more time points [27]. However, there is also a risk of over-adjustment in such an analysis, as the hostile environment policies may have acted through many variables to impact mental health. We therefore chose not to control for particular time-varying socioeconomic variables, notably income and employment, because they were assumed to be on the causal pathway as mediators in the relationship between the hostile environment policies and mental health; some of the hostile environment policies had a direct impact on employment for minoritised ethnic groups by increasing discriminatory “right to employment” checks, so adjusting for employment would bias our estimates of the mental health impact.

Overall, our findings provide evidence of a differential impact of changing immigration policies on the mental health of ethnic groups in the UK. Further, they underline the importance of disaggregating minoritised ethnic groups and highlight a potential impact of hostile environment policies on Pakistani and Bangladeshi groups in the UK.

## Supplementary Information

Below is the link to the electronic supplementary material.

Supplementary file1 (DOCX 35 KB)

Supplementary file2 (DOCX 29 KB)

Supplementary file3 (DOCX 42 KB)

## Data Availability

Understanding Society data are available through the UK Data Service. Researchers who would like to use Understanding Society need to register with the UK Data Service before being allowed to download datasets.
