# Supplementary material for: Exploring the impact of ‘hostile environment’ policies on psychological distress of ethnic groups in the UK: a differences-in-differences analysis
Source: Soc Psychiatry Psychiatr Epidemiol. 2024 Jul 8;60(1):139–48. doi: 10.1007/s00127-024-02705-2 (PMC11790676; doi:10.1007/s00127-024-02705-2)
Supplement: Supplementary file 1 — Supplementary file1 (DOCX 35 KB) [file 127_2024_2705_MOESM1_ESM.docx]

Our sample included all adults (over age 16) who had ethnicity information and who had participated in Wave 1 of Understanding Society. There are two types of missing data in this study – wave non-response and item non-response.

**Wave non-response:** As with many longitudinal studies, some study participants from Wave 1 did not respond in all subsequent study waves. 28.8% of the sample participated in all 10 waves while 9.1% missed a single wave. Table A1 summarises the number of participants who completed an interview in each wave. In each subsequent wave, a smaller proportion of the original sample responded: 75.2% in Wave 2 and only 38.3% in Wave 10. We estimated inverse probability weights to account for differential non-response, as reported in our weighting strategy.

**Table A1 Wave respondents and non-respondents**

| **Era** | **Wave** | **Respondents** | | **Non-respondents** | |
| --- | --- | --- | --- | --- | --- |
|  |  | **n** | **%** | **n** | **%** |
| Pre-policy era | Wave 1 | 42,968 | 100.0 | 0 | 0.0 |
|  | Wave 2 | 32,314 | 75.2 | 10,654 | 24.8 |
|  | Wave 3 | 28,106 | 65.4 | 14,862 | 65.4 |
| Transition era | Wave 4 | 25,594 | 59.6 | 17,374 | 40.4 |
|  | Wave 5 | 23,759 | 55.3 | 19,209 | 44.7 |
|  | Wave 6 | 21,106 | 49.1 | 21,862 | 50.9 |
| Ongoing policy era | Wave 7 | 19,980 | 46.5 | 22,988 | 53.5 |
|  | Wave 8 | 18,778 | 43.7 | 24,190 | 56.3 |
|  | Wave 9 | 17,275 | 40.2 | 25,693 | 59.8 |
|  | Wave 10 | 16,455 | 38.3 | 26,513 | 61.7 |

**Item non-response:** Key variables, including ethnicity, sex, and age were complete for all participants, but there was missing information for confounders (marital status, education, citizenship status, urban/rural status) and outcomes (psychological distress). Table A2 summarises wave-specific item non-response for marital status, education, citizenship status, urban/rural status, and psychological distress among those who participated in that study wave. We used multiple imputation with chained equations, estimating 100 imputed datasets. Our primary results were generated from the imputed data.

**Table A2 Item non-response among respondents in each wave**

| **Wave** | **Total** | **Marital status** | | **Education** | | **Citizenship status** | | **Urban/ rural status** | | **Psychological distress** | |
| --- | --- | --- | --- | --- | --- | --- | --- | --- | --- | --- | --- |
|  | **n** | **n** | **%** | **n** | **%** | **n** | **%** | **n** | **%** | **n** | **%** |
| Wave 1 | 42,968 | 14 | 0.0 | 32 | 0.1 | 9 | 0.0 | 0 | 0.0 | 6,925 | 16.1 |
| Wave 2 | 32,314 | 3 | 0.0 | 21 | 0.1 | 6 | 0.0 | 20 | 0.1 | 4,527 | 14.0 |
| Wave 3 | 28,106 | 2 | 0.0 | 14 | 0.0 | 6 | 0.0 | 3 | 0.0 | 3,128 | 11.1 |
| Wave 4 | 25,594 | 64 | 0.3 | 16 | 0.1 | 7 | 0.0 | 8 | 0.0 | 2,378 | 9.3 |
| Wave 5 | 23,759 | 37 | 0.2 | 12 | 0.1 | 7 | 0.0 | 10 | 0.0 | 2,273 | 9.6 |
| Wave 6 | 21,106 | 53 | 0.3 | 10 | 0.0 | 5 | 0.0 | 6 | 0.0 | 1,054 | 5.0 |
| Wave 7 | 19,980 | 28 | 0.1 | 10 | 0.1 | 5 | 0.0 | 4 | 0.0 | 823 | 4.1 |
| Wave 8 | 18,778 | 15 | 0.1 | 9 | 0.0 | 5 | 0.0 | 10 | 0.1 | 743 | 4.0 |
| Wave 9 | 17,275 | 22 | 0.1 | 7 | 0.0 | 4 | 0.0 | 11 | 0.1 | 683 | 4.0 |
| Wave 10 | 16,455 | 40 | 0.2 | 7 | 0.0 | 3 | 0.0 | 9 | 0.1 | 654 | 4.0 |

**Comparing characteristics of participants with complete data vs. missing data:** We compared the characteristics of those in the complete-case sample (n=36,007) with those missing data on the outcome or covariates (n=6,961) (Table A3).

There were differences across most demographic characteristics. Participants from minoritised ethnic groups were more likely to have missing data compared to White British participants. Participants with lower education, non-British citizens, those living in urban areas, and participants who were separated, divorced, widowed, or single all had higher odds of having missing data when compared to the respective reference categories (Table A3).

**Table A3 Sample characteristics of participants with complete data (n=36,007) and participants with missing data (n=6,961)**

| **Variable** | **Complete cases** | | **Missing^*^** | | **Regression** | | | |
| --- | --- | --- | --- | --- | --- | --- | --- | --- |
|  | **n** | **%** | **n** | **%** | **OR** | **95%** | **CI** | **p-value** |
| **Ethnicity** |  |  |  |  |  |  |  | <0.001 |
| African | 945 | 2.6 | 493 | 7.1 | 3.60 | 3.22 | 4.04 |  |
| Bangladeshi | 621 | 1.7 | 511 | 7.3 | 5.68 | 5.04 | 6.42 |  |
| Caribbean | 797 | 2.2 | 340 | 4.9 | 2.95 | 2.59 | 3.36 |  |
| Indian | 1,363 | 3.8 | 542 | 7.8 | 2.75 | 2.47 | 3.05 |  |
| Pakistani | 905 | 2.5 | 533 | 7.7 | 4.07 | 3.64 | 4.55 |  |
| White British (ref.) | 31,376 | 87.1 | 4,542 | 65.3 | 1 |  |  |  |
| **Sex** |  |  |  |  |  |  |  | 0.25 |
| Female (ref.) | 20,163 | 56.0 | 3,846 | 55.3 | 1 |  |  |  |
| Male | 15,844 | 44.0 | 3,115 | 44.8 | 1.03 | 0.98 | 1.09 |  |
| **Education** |  |  |  |  |  |  |  | <0.001 |
| A-levels or higher (ref.) | 14,975 | 41.6 | 2,336 | 33.6 | 1 |  |  |  |
| GCSE or lower | 21,032 | 58.4 | 4,593 | 66.0 | 1.40 | 1.33 | 1.48 |  |
| Missing |  |  | 32 | 0.5 | 1 | . | . |  |
| **British citizen** |  |  |  |  |  |  |  | <0.001 |
| Yes (ref.) | 34,863 | 96.8 | 6,347 | 91.2 | 1 | - | - |  |
| No | 1,144 | 3.2 | 605 | 8.7 | 2.90 | 2.62 | 3.22 |  |
|  |  |  | 9 | 0.1 |  |  |  |  |
| **Urban/rural** |  |  |  |  |  |  |  | <0.001 |
| Urban | 27,760 | 77.1 | 5,884 | 84.5 | 1.62 | 1.51 | 1.74 |  |
| Rural (ref.) | 8,247 | 22.9 | 1,077 | 15.5 | 1 |  |  |  |
| **Marital status** |  |  |  |  |  |  |  | <0.001 |
| Partnered/ married (ref.) | 22,735 | 63.1 | 4,063 | 58.4 | 1 |  |  |  |
| Separated / divorced / widowed | 5,353 | 14.9 | 1,310 | 18.8 | 1.37 | 1.28 | 1.47 |  |
| Single | 7,919 | 22.0 | 1,574 | 22.6 | 1.11 | 1.04 | 1.18 |  |
| Missing |  |  | 14 | 0.2 | 1 | . | . |  |
|  |  |  |  |  |  |  |  |  |
|  | **Mean** | **SD** | **Mean** | **SD** | **Coeff.** | **95%** | **CI** | **p-value** |
| **Age** |  |  |  |  |  |  |  | <0.001 |
| Mean (SD) | 46.56 | 18.04 | 47.75 | 19.27 | 0.00 | 0.00 | 0.00 |  |
| **Psychological distress** |  |  |  |  |  |  |  | 0.03 |
| GHQ-12 mean (SD) | 1.76 | 2.88 | 2.78 | 3.30 | 0.00 | 0.00 | 0.00 |  |

*^*^ Missing included participants with missing information on any of the following variables: sex, age, marital status, education, citizenship status, urban/rural status, GHQ-12 score*

*n: number; SD: standard deviation; GHQ: General Health Questionnaire; Ref: reference category; Coef: coefficient*
