# Supplementary material for: Exploring the impact of ‘hostile environment’ policies on psychological distress of ethnic groups in the UK: a differences-in-differences analysis
Source: Soc Psychiatry Psychiatr Epidemiol. 2024 Jul 8;60(1):139–48. doi: 10.1007/s00127-024-02705-2 (PMC11790676; doi:10.1007/s00127-024-02705-2)
Supplement: Supplementary file 2 — Supplementary file2 (DOCX 29 KB) [file 127_2024_2705_MOESM2_ESM.docx]

**Table C1 Mean psychological distress (GHQ-12) score by ethnicity and study wave**

|  |  | **African** | | **Bangladeshi** | | **Caribbean** | | **Indian** | | **Pakistani** | | **White British** | | |
| --- | --- | --- | --- | --- | --- | --- | --- | --- | --- | --- | --- | --- | --- | --- |
| **Era** | **Wave** | **Mean** | **SD** | **Mean** | **SD** | **Mean** | **SD** | **Mean** | **SD** | **Mean** | **SD** | **Mean** | **SD** |  |
| Pre-policy era | Wave 1 | 1.86 | 2.81 | 2.24 | 2.94 | 2.01 | 2.95 | 1.78 | 2.85 | 2.22 | 3.19 | 1.73 | 2.87 |  |
|  | Wave 2 | 1.87 | 2.89 | 2.31 | 3.13 | 2.13 | 3.24 | 1.91 | 3.11 | 2.43 | 3.40 | 1.74 | 3.00 |  |
|  | Wave 3 | 2.01 | 2.90 | 2.18 | 3.05 | 2.17 | 3.20 | 1.86 | 3.01 | 2.72 | 3.49 | 1.74 | 2.96 |  |
| Transition era | Wave 4 | 1.64 | 2.75 | 2.42 | 3.23 | 2.06 | 3.19 | 1.76 | 2.96 | 2.49 | 3.49 | 1.71 | 2.99 |  |
|  | Wave 5 | 1.57 | 2.81 | 2.39 | 3.41 | 2.04 | 3.16 | 1.80 | 2.97 | 2.73 | 3.73 | 1.79 | 3.02 |  |
|  | Wave 6 | 1.70 | 2.73 | 2.02 | 3.28 | 1.96 | 3.20 | 1.79 | 2.90 | 2.70 | 3.52 | 1.58 | 2.85 |  |
| Ongoing policy era | Wave 7 | 1.42 | 2.48 | 2.37 | 3.52 | 2.10 | 3.30 | 1.93 | 3.26 | 2.30 | 3.39 | 1.63 | 2.92 |  |
|  | Wave 8 | 1.62 | 2.67 | 1.87 | 2.94 | 2.21 | 3.49 | 1.72 | 3.14 | 2.47 | 3.75 | 1.69 | 3.00 |  |
|  | Wave 9 | 1.81 | 3.01 | 2.14 | 3.24 | 2.16 | 3.50 | 1.64 | 3.03 | 2.20 | 3.47 | 1.69 | 3.02 |  |
|  | Wave 10 | 2.02 | 3.15 | 2.10 | 3.14 | 1.76 | 2.94 | 1.58 | 2.95 | 2.35 | 3.57 | 1.70 | 3.00 |  |

**Figure C1 Mean psychological distress (GHQ-12) score by ethnicity and study wave**
