# Supplementary material for: Exploring the impact of ‘hostile environment’ policies on psychological distress of ethnic groups in the UK: a differences-in-differences analysis
Source: Soc Psychiatry Psychiatr Epidemiol. 2024 Jul 8;60(1):139–48. doi: 10.1007/s00127-024-02705-2 (PMC11790676; doi:10.1007/s00127-024-02705-2)
Supplement: Supplementary file 3 — Supplementary file3 (DOCX 42 KB) [file 127_2024_2705_MOESM3_ESM.docx]

As a sensitivity analysis, we restricted the sample to complete cases (n=36,007) to examine if the patterns of psychological distress by ethnic group were similar to those described in the imputed analysis. Table B1 summarises the sample characteristics at Wave 1 (2009-10) for the participants included in the complete case analysis, who had complete information on for psychological distress and all covariates (sex, age, education, citizenship status, urban/rural status, and marital status)

**Table B1 Sample characteristics by ethnic group in Wave 1, complete cases (n=36,007)**

|  | **African** | | **Bangladeshi** | | **Caribbean** | | **Indian** | | **Pakistani** | | **White British** | | |
| --- | --- | --- | --- | --- | --- | --- | --- | --- | --- | --- | --- | --- | --- |
|  | **n** | **%** | **n** | **%** | **n** | **%** | **n** | **%** | **n** | **%** | **n** | **%** |  |
| **Total** |  |  |  |  |  |  |  |  |  |  |  |  |  |
| n (% of total sample) | 945 | 2.6 | 621 | 1.7 | 797 | 2.2 | 1,363 | 3.8 | 905 | 2.5 | 31,376 | 87.1 |  |
| **Sex** |  |  |  |  |  |  |  |  |  |  |  |  |  |
| Female | 532 | 56.3 | 306 | 49.3 | 499 | 62.6 | 660 | 48.4 | 459 | 50.7 | 17,707 | 56.4 |  |
| Male | 413 | 43.7 | 315 | 50.7 | 298 | 37.4 | 703 | 51.6 | 446 | 49.3 | 13,669 | 43.6 |  |
| **Age** |  |  |  |  |  |  |  |  |  |  |  |  |  |
| Mean (SD) | 35.3 | 12.4 | 31.2 | 11.4 | 43.9 | 16.0 | 38.0 | 14.6 | 34.2 | 13.2 | 48.0 | 18.1 |  |
| **Education** |  |  |  |  |  |  |  |  |  |  |  |  |  |
| A levels or higher | 621 | 65.7 | 291 | 46.9 | 335 | 42.0 | 911 | 66.8 | 465 | 51.4 | 12,352 | 39.4 |  |
| GCSE or lower | 324 | 34.3 | 330 | 53.1 | 462 | 58.0 | 452 | 33.2 | 440 | 48.6 | 19,024 | 60.6 |  |
| **British citizen** |  |  |  |  |  |  |  |  |  |  |  |  |  |
| Yes | 548 | 58.0 | 506 | 81.5 | 735 | 92.2 | 987 | 72.4 | 758 | 83.8 | 31,329 | 99.8 |  |
| No | 397 | 42.0 | 115 | 18.5 | 62 | 7.8 | 376 | 27.6 | 147 | 16.2 | 47 | 0.2 |  |
| **Urban/rural** |  |  |  |  |  |  |  |  |  |  |  |  |  |
| Urban | 938 | 99.3 | 616 | 99.2 | 793 | 99.5 | 1,339 | 98.2 | 900 | 99.4 | 23,174 | 73.9 |  |
| Rural | 7 | 0.7 | 5 | 0.8 | 4 | 0.5 | 24 | 1.8 | 5 | 0.6 | 8,202 | 26.1 |  |
| **Marital status** |  |  |  |  |  |  |  |  |  |  |  |  |  |
| Partnered / married / cohabiting) | 449 | 47.5 | 357 | 57.5 | 287 | 36.0 | 888 | 65.2 | 577 | 63.8 | 20,177 | 64.3 |  |
| Separated / divorced / widowed | 112 | 11.8 | 37 | 6.0 | 152 | 19.1 | 70009 | 6.7 | 700 | 7.7 | 4,891 | 15.6 |  |
| Single | 384 | 40.6 | 227 | 36.6 | 358 | 44.9 | 384 | 28.2 | 258 | 28.5 | 6,308 | 20.1 |  |
| **Psychological distress (GHQ-12)** |  |  |  |  |  |  |  |  |  |  |  |  |  |
| Mean (SD) | 1.86 | 2.81 | 2.23 | 2.93 | 2.01 | 2.95 | 1.78 | 2.85 | 2.23 | 3.19 | 1.73 | 2.87 |  |

*n: number; SD: standard deviation*

**Table B2 Unadjusted and adjusted^1^ regression, complete cases (n=36,007)**

|  | | **UNADJUSTED** | | | | **ADJUSTED^1^** | | | |
| --- | --- | --- | --- | --- | --- | --- | --- | --- | --- |
|  | | **Coeff.** | **95% CI** | | **p-value** | **Coeff.** | **95% CI** | | **p-value** |
| **Era** | |  |  |  |  |  |  |  |  |
| Pre-policy era (ref.) | | 0.00 |  |  |  | 0.00 |  |  |  |
| Transition era | | 0.00 | -0.03 | 0.03 | 0.90 | 0.01 | -0.02 | 0.04 | 0.37 |
| Ongoing policy era | | 0.02 | -0.01 | 0.06 | 0.24 | 0.05 | 0.02 | 0.09 | 0.00 |
| **Ethnicity** | |  |  |  |  |  |  |  |  |
| African | | 0.12 | -0.04 | 0.28 | 0.13 | 0.00 | -0.18 | 0.17 | 0.97 |
| Bangladeshi | | 0.39 | 0.18 | 0.60 | 0.00 | 0.31 | 0.10 | 0.53 | 0.00 |
| Caribbean | | 0.31 | 0.12 | 0.50 | 0.00 | 0.16 | -0.04 | 0.35 | 0.11 |
| Indian | | 0.03 | -0.11 | 0.17 | 0.66 | 0.00 | -0.15 | 0.15 | 1.00 |
| Pakistani | | 0.63 | 0.44 | 0.83 | 0.00 | 0.56 | 0.36 | 0.77 | 0.00 |
| White British (ref.) | |  |  |  |  |  |  |  |  |
| **Interaction** | |  |  |  |  |  |  |  |  |
| **Era** | **Ethnicity** |  |  |  |  |  |  |  |  |
| Transition | African | -0.31 | -0.54 | -0.08 | 0.01 | -0.31 | -0.54 | -0.08 | 0.01 |
|  | Bangladeshi | 0.11 | -0.24 | 0.47 | 0.53 | 0.10 | -0.25 | 0.46 | 0.57 |
|  | Caribbean | -0.10 | -0.34 | 0.14 | 0.42 | -0.11 | -0.35 | 0.12 | 0.34 |
|  | Indian | -0.22 | -0.41 | -0.04 | 0.02 | -0.23 | -0.41 | -0.04 | 0.02 |
|  | Pakistani | 0.01 | -0.26 | 0.29 | 0.92 | 0.01 | -0.26 | 0.29 | 0.92 |
| Ongoing | African | -0.36 | -0.61 | -0.11 | 0.01 | -0.35 | -0.60 | -0.10 | 0.01 |
|  | Bangladeshi | 0.11 | -0.39 | 0.62 | 0.66 | 0.11 | -0.40 | 0.61 | 0.68 |
|  | Caribbean | -0.33 | -0.63 | -0.03 | 0.03 | -0.36 | -0.65 | -0.06 | 0.02 |
|  | Indian | -0.25 | -0.46 | -0.05 | 0.01 | -0.24 | -0.45 | -0.04 | 0.02 |
|  | Pakistani | -0.23 | -0.56 | 0.10 | 0.17 | -0.22 | -0.56 | 0.11 | 0.19 |
| **Constant** | |  |  |  |  |  |  |  |  |
| Fixed effect | | 1.74 | 1.72 | 1.77 | 0.00 | 2.01 | 1.92 | 2.10 | 0.00 |
| **Random-effects parameters^3^** | |  |  |  | **RSE** |  |  |  | **RSE** |
| Individual-level variance | | 4.83 | 4.71 | 4.96 | 0.07 | 4.72 | 4.59 | 4.82 | 0.06 |
| Variance of the residuals (variance within individuals) | | 4.39 | 4.31 | 4.47 | 0.04 | 4.39 | 4.31 | 4.47 | 0.04 |

*^1^Adjusted for sex, age, marital status, education, citizenship status, urban/rural status.  ^2^100 imputed datasets; ^3^Mixed models with random effects to account for clustering within each individual over time. Coef: coefficient; CI: confidence interval; Ref: reference category; RSE: robust standard error*

**Table B2 Marginal mean psychological distress scores by ethnic group and policy era, complete cases (n=36,007)**

|  | **PRE-POLICY ERA** | | | | **TRANSITION ERA** | | | | **ONGOING POLICY ERA** | | | |
| --- | --- | --- | --- | --- | --- | --- | --- | --- | --- | --- | --- | --- |
|  | **Coeff.** | **95 %** | **CI** |  | **Coeff.** | **95 %** | **CI** |  | **Coeff.** | **95 %** | **CI** |  |
| **African** |  |  |  |  |  |  |  |  |  |  |  |  |
| Unadjusted | 1.87 | 1.71 | 2.02 |  | 1.56 | 1.34 | 1.78 |  | 1.53 | 1.29 | 1.77 |  |
| Adjusted^1^ | 1.71 | 1.54 | 1.88 |  | 1.41 | 1.18 | 1.65 |  | 1.42 | 1.17 | 1.66 |  |
| **Bangladeshi** |  |  |  |  |  |  |  |  |  |  |  |  |
| Unadjusted | 2.14 | 1.93 | 2.34 |  | 2.25 | 1.90 | 2.60 |  | 2.27 | 1.82 | 2.72 |  |
| Adjusted^1^ | 2.03 | 1.81 | 2.24 |  | 2.14 | 1.79 | 2.50 |  | 2.19 | 1.73 | 2.64 |  |
| **Caribbean** |  |  |  |  |  |  |  |  |  |  |  |  |
| Unadjusted | 2.06 | 1.87 | 2.25 |  | 1.96 | 1.72 | 2.19 |  | 1.75 | 1.46 | 2.03 |  |
| Adjusted^1^ | 1.87 | 1.68 | 2.06 |  | 1.77 | 1.53 | 2.01 |  | 1.57 | 1.29 | 1.85 |  |
| **Indian** |  |  |  |  |  |  |  |  |  |  |  |  |
| Unadjusted | 1.77 | 1.64 | 1.91 |  | 1.55 | 1.37 | 1.73 |  | 1.54 | 1.35 | 1.73 |  |
| Adjusted^1^ | 1.71 | 1.57 | 1.86 |  | 1.50 | 1.31 | 1.69 |  | 1.52 | 1.33 | 1.72 |  |
| **Pakistani** |  |  |  |  |  |  |  |  |  |  |  |  |
| Unadjusted | 2.38 | 2.18 | 2.57 |  | 2.39 | 2.12 | 2.65 |  | 2.16 | 1.86 | 2.47 |  |
| Adjusted^1^ | 2.28 | 2.08 | 2.48 |  | 2.30 | 2.04 | 2.57 |  | 2.11 | 1.80 | 2.42 |  |
| **White British** |  |  |  |  |  |  |  |  |  |  |  |  |
| Unadjusted | 1.74 | 1.72 | 1.77 |  | 1.74 | 1.71 | 1.77 |  | 1.76 | 1.73 | 1.80 |  |
| Adjusted^1^ | 1.71 | 1.69 | 1.74 |  | 1.73 | 1.69 | 1.76 |  | 1.77 | 1.73 | 1.81 |  |

^1^*Adjusted for sex, age, marital status, education, citizenship status, urban/rural status*

*Coef: coefficient; CI: confidence interval*
